# Case Report: Till death - or the urologist - do us part: management of penile wedding-ring incarceration

**DOI:** 10.3389/fruro.2026.1767585

**Published:** 2026-04-29

**Authors:** Jaisukh Kalathia, Bharti Talreja, Kaushal Patel, Arvind Valiya, Giriraj Vala, Ayush Khetarpal

**Affiliations:** 1 Department of Urology, Fortune Urology Clinic, Botad, India; 2 Department of Urology, Nephron Kidney Hospital, Anand, India; 3 Department of Urology, Valiya Kidney Hospital, Bhavnagar, India; 4 Department of Urology, KIMS, Ahmedabad, India; 5 Department of Urology, Khetarpal Hospital, New Delhi, India

**Keywords:** erectile desfunction (ED), incarceration, orthopaedic cutter, penile strangulation, wedding ring

## Abstract

Penile strangulation is a rare urological emergency that can rapidly progress to vascular compromise and irreversible tissue damage. Metallic constricting devices pose the greatest challenge due to their rigidity and resistance to conventional removal techniques. We report the case of an adult male who developed progressive penile swelling and pain after self-application of a metallic wedding ring during an episode of emotional distress, necessitating mechanical sectioning with a tungsten-carbide orthopaedic cutter under continuous protective shielding for safe removal.

## Introduction

Penile constriction by a foreign device is an uncommon yet potentially devastating urological emergency. Constricting objects are generally applied to enhance sexual performance, prolong erections, or delay ejaculation, and may occasionally be associated with underlying psychological disorders. Prolonged application impedes venous and lymphatic drainage, resulting in progressive oedema, pain, and vascular compromise of the distal penis. If untreated, the condition carries a high risk of severe complications, including ischemia, necrosis, gangrene, and auto-amputation (1). Presentation is often delayed due to embarrassment, by which time features of ischemia may already be established. The nature of the constricting material plays a crucial role in determining extraction difficulty, with metallic devices posing the greatest challenge because of their rigidity and resistance to conventional cutting tools. Reported objects include industrial metal rings, nuts, and sprockets, which may require specialized equipment for removal. The rarity and heterogeneity of these cases mean that there is no standardized management protocol, and emergency departments frequently lack the tools or experience needed for timely intervention. Early and individualized management is essential to preserve erectile function and avoid irreversible penile injury. We present a rare case of penile strangulation by a wedding ring and outline the stepwise surgical approach required for successful penile salvage.

## Case

A 38-year-old male presented to the urological emergency department with complaints of penile pain and swelling. According to the patient, he had placed his engagement ring at the base of his penis following a disagreement that led to the cancellation of his marriage. This event had occurred approximately 24 hours prior to hospital presentation. After placing the ring, he consumed excessive alcohol, fell asleep, and forgot to remove it. On waking the following afternoon, he experienced significant pain and attempted multiple times to remove the ring unsuccessfully. Feeling frightened and too embarrassed to inform his relatives, he tried various methods to remove it by himself. He observed progressive pain and gradually increase in swelling distal to the constricting ring at the penile base. Despite this, he could void urine without much difficulty.

**Figure 1:**
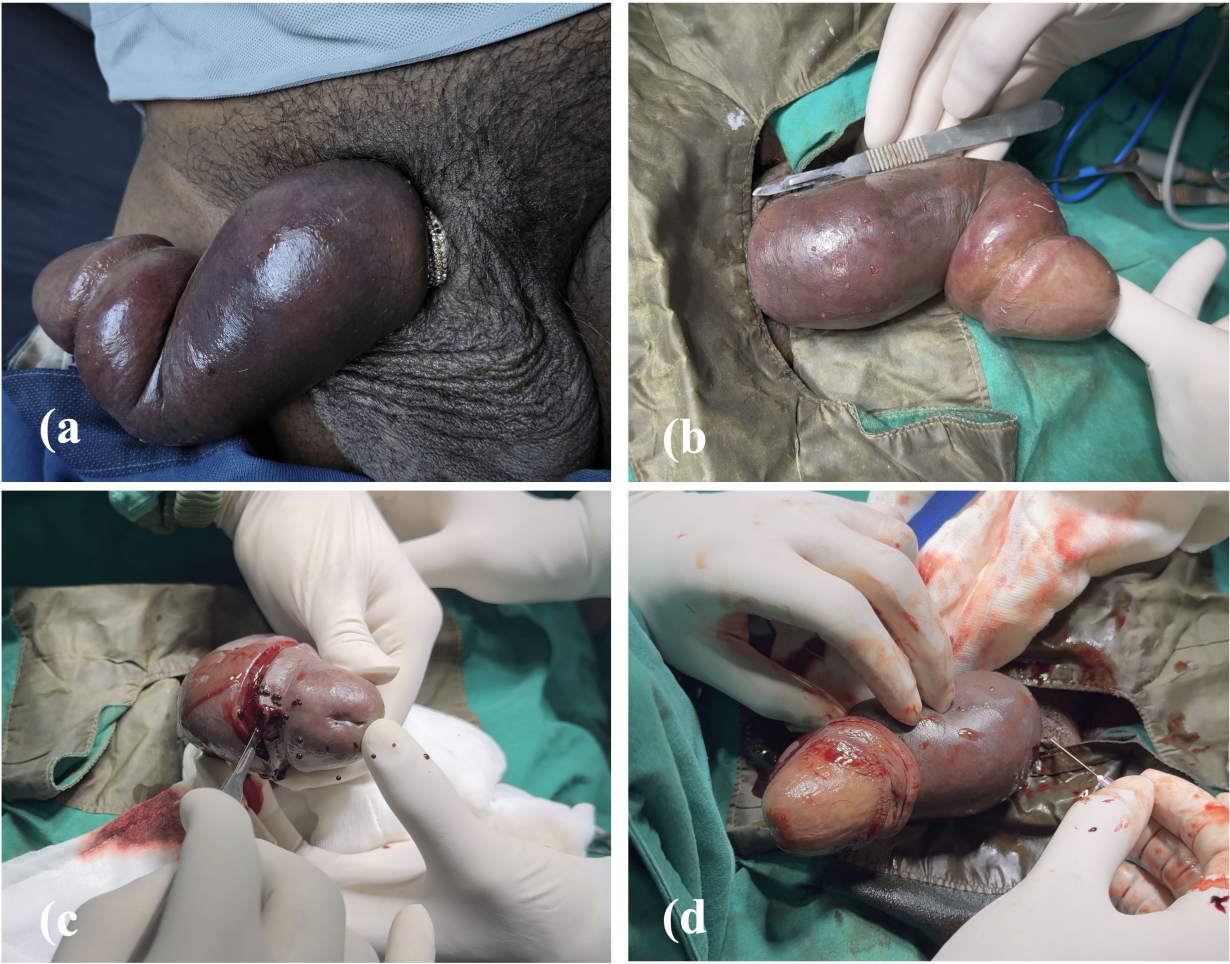
**(a)** Ring constricting penile base **(b)** Penile shaft oedematous and engorged **(c)** Circumcoronal incision **(d)** Multiple needle punctures.

On examination, a 1 cm-wide, 2 mm-thick stainless-steel ring was found tightly constricting the penile base. (**Figure 1a**) The shaft was markedly oedematous and engorged, with venous congestion and cyanotic discoloration distal to the constriction. After evaluating available management options, the patient was taken for intervention under general anaesthesia. (**Figure 1b**) A circumcoronal incision was made for oedema decompression, (**Figure 1c**) supplemented by multiple needle punctures over the shaft and penile base to drain the trapped fluid and prevent impending compartment syndrome. (**Figure 1d**) Although these manoeuvres resulted in successful detumescence, the ring remained firmly in place.

**Figure 2:**
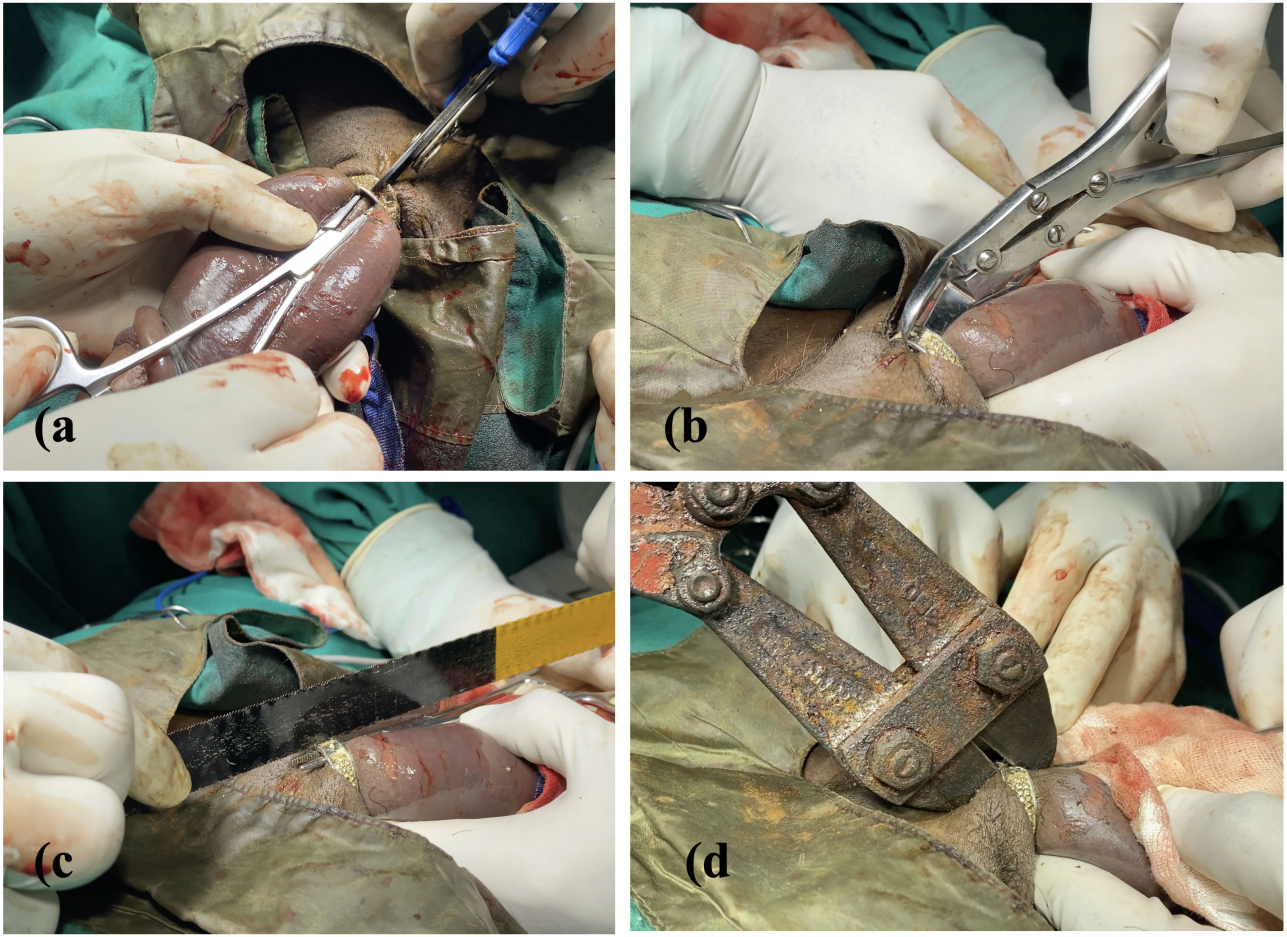
**(a)** Surgical stapler-pin cutter **(b)** Bone cutter **(c)** Hacksaw **(d)** An orthopaedic TC (Tungsten Carbide) Tip cutter.

A stepwise escalation of mechanical removal techniques was undertaken using instruments immediately accessible in the emergency setting. An attempt to section the ring using a surgical stapler-pin cutter (**Figure 2a**) was unsuccessful, followed by a trial with a heavy bone cutter, during which an artery forceps was inserted between the penile skin and ring to prevent inadvertent injury. (**Figure 2b**) As neither instrument produced any effect on the metal, a hacksaw—designed for fine metal cutting—was tried but again failed. (**Figure 2c**).

**Figure 3:**
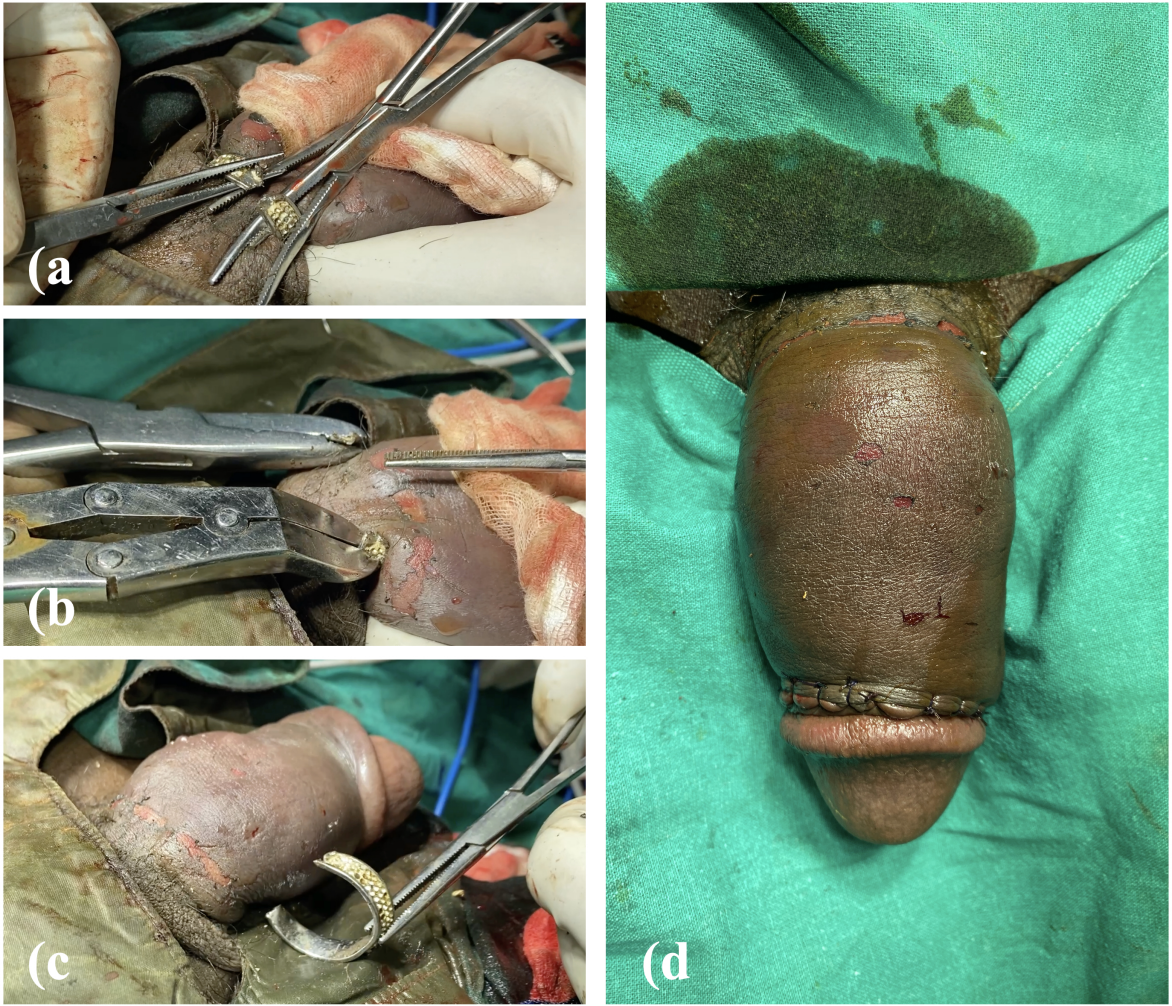
**(a)** opening cut ends of ring using artery forceps **(b)** Bone cutters applied laterally **(c)** Ring disengaged **(d)** Detumescence and circumcoronal incision sutured.

Finally, an orthopaedic TC (Tungsten Carbide) Tip Cutter, engineered to cut high-resistance metallic implants such as wires and screws, was employed. With considerable force, the TC cutter successfully divided the ring. (**Figure 2d**) Attempts to try open the cut ends using artery forceps (**Figure 3a**) were unsuccessful, necessitating the simultaneous use of two bone cutters applied laterally (**Figure 3b**) to spread the ring until it disengaged completely from the penis. (**Figure 3c**) Following removal, the cyanotic discoloration resolved promptly. (**Figure 3d**) The circumcoronal incision was sutured, and the patient was shifted to recovery in a haemodynamically stable condition.

## Discussion

Penile strangulation with metallic objects is an uncommon urological emergency that warrants immediate intervention to prevent permanent structural and functional damage. Delayed presentation is not unusual, as patients frequently attempt self-removal and may hesitate to seek medical care due to embarrassment (2). Motivations for applying constricting devices include sexual experimentation, non-medical attempts to improve erectile function, and underlying psychological stressors. A wide spectrum of foreign objects has been documented, and management difficulty varies with material rigidity and accessibility; metallic rings pose the greatest challenge.

Prolonged constriction initiates a pathophysiological sequence beginning with venous obstruction and lymphatic stasis, followed by progressive oedema and elevated intracorporeal pressure that ultimately compromises arterial inflow (3). If untreated, this cascade may lead to ischemic necrosis, gangrene, and auto-amputation. Prompt recognition and early decompression are therefore crucial to halt tissue injury and preserve penile viability. In our case, there was only superficial skin abrasion, and no necrosis occurred.

Management must be tailored to the presenting object and degree of oedema. A systematic approach includes a detailed history of the object and duration of incarceration, followed by focussed genital examination to assess vascularity, sensation, and tissue viability. Imaging is generally unnecessary in the acute setting. Initial decompression of distal oedema using multiple needle punctures or a circumcoronal incision often facilitates removal by restoring penile flaccidity (4). In our case, despite successful detumescence, the metallic ring (titanium composition) remained fixed, necessitating mechanical cutting with an orthopaedic tungsten-carbide cutter.

When cutting is required, protective shielding—such as a laryngoscope blade, tongue depressor, or PVC plaque—should be placed between the device and the penile shaft to minimise thermal and mechanical injury. After removal, careful inspection of the penis is essential to identify devitalised areas and prevent complications such as infection, urethral fistula, fibrosis, erectile dysfunction, and penile necrosis. Long-term follow-up is recommended, and psychological assessment should be considered when behavioural or emotional triggers are suspected. In our patients a psychological assessment was performed, and the patient was evaluated by a psychiatrist who advised antidepressants. In addition, a psychosexual assessment was also conducted as part of a comprehensive approach to care and recurrence prevention. At 3-month follow-up, the patient demonstrated an International Index of Erectile Function (IIEF) score of 23, with preserved sexual activity. He reported no lower urinary tract symptoms, was voiding well with a normal uroflowmetry pattern, and therefore a retrograde urethrogram was deemed unnecessary.

## Conclusion

Penile strangulation with metallic objects constitutes a rare but definitive urological emergency requiring prompt, structured intervention. Early decompression to relieve distal oedema is pivotal for successful device removal and prevention of irreversible ischemic injury. When metallic devices mandate heavy-duty cutting tools, meticulous tissue protection coupled with post-extraction surveillance is essential to minimise iatrogenic harm and detect late complications.

## Data Availability

The original contributions presented in the study are included in the article/Supplementary Material. Further inquiries can be directed to the corresponding author.
